# ADAR1 suppression causes interferon signaling and transposable element transcript accumulation in human astrocytes

**DOI:** 10.3389/fnmol.2023.1263369

**Published:** 2023-10-25

**Authors:** Cali M. McEntee, Alyssa N. Cavalier, Thomas J. LaRocca

**Affiliations:** ^1^Department of Health and Exercise Science, Colorado State University, Fort Collins, CO, United States; ^2^Center for Healthy Aging, Colorado State University, Fort Collins, CO, United States

**Keywords:** ADAR1, double-stranded RNA, transposable elements, astrocytes, transcriptomics, neuroinflammation

## Abstract

Neuroinflammation is a central mechanism of brain aging and Alzheimer’s disease (AD), but the exact causes of age- and AD-related neuroinflammation are incompletely understood. One potential modulator of neuroinflammation is the enzyme adenosine deaminase acting on RNA 1 (ADAR1), which regulates the accumulation of endogenous double-stranded RNA (dsRNA), a pro-inflammatory/innate immune activator. However, the role of ADAR1 and its transcriptomic targets in astrocytes, key mediators of neuroinflammation, have not been comprehensively investigated. Here, we knock down ADAR1 in primary human astrocytes via siRNA transfection and use transcriptomics (RNA-seq) to show that this results in: (1) increased expression of type I interferon and pro-inflammatory signaling pathways and (2) an accumulation of transposable element (TE) transcripts with the potential to form dsRNA. We also show that our findings may be clinically relevant, as *ADAR1* gene expression declines with brain aging and AD in humans, and this is associated with a similar increase in TE transcripts. Together, our results suggest an important role for ADAR1 in preventing pro-inflammatory activation of astrocytes in response to endogenous dsRNA with aging and AD.

## Introduction

Neuroinflammation is a major mechanism of brain aging and age-related neurodegenerative disorders like Alzheimer’s disease (AD), and it is characterized by pro-inflammatory activation of glial cells (Colombo and Farina, 2016; Giovannoni and Quintana, 2020). Astrocytes, the most common glial cells in the brain, interface closely with neurons and other central nervous system (CNS) cells (Nedergaard et al., 2003), and pro-inflammatory astrocytes have been documented in both acute neuroinflammation and chronic, age-related neurodegenerative diseases like AD (Farina et al., 2007; Burda et al., 2016; Habib et al., 2020). However, the upstream causes of pro-inflammatory activation in astrocytes during aging and/or AD are not fully understood.

One biologically important, intracellular driver of pro-inflammatory signaling is double-stranded RNA (dsRNA), which is a virus-associated molecular pattern. Cytoplasmic dsRNA binds to innate immune sensors that stimulate type I interferon and pro-inflammatory signaling pathways, a normal response to dsRNA virus infections (Gantier and Williams, 2007). However, cytoplasmic dsRNA may also originate from endogenous sources (e.g., genomic DNA/RNA). A key regulator of endogenous dsRNA is the enzyme adenosine deaminase acting on RNA 1 (ADAR1) (Hogg et al., 2011), which regulates dsRNA through adenosine-to-inosine (A-to-I) editing that disrupts base-pairing to prevent the accumulation of dsRNA in the cytoplasm (George et al., 2016; Solomon et al., 2017). ADAR1 is important in reducing type I interferon and pro-inflammatory signaling (Lamers et al., 2019; Samuel, 2019), and although *Adar1* (mouse ortholog) knockout or editing inhibition in mice is embryonically lethal, this can be prevented by simultaneous knockout of the dsRNA sensor melanoma differentiation-associated protein 5 (MDA5) (Liddicoat et al., 2015; Kim et al., 2021; Guo et al., 2022b). Recent data in *Drosophila* also show that catalytically inactive *Adar* (fly ortholog) causes innate immune activation in brain-like tissue (Deng et al., 2020), and mutations in human *ADAR1* result in Aicardi Goutières syndrome (AGS), which is characterized by type I interferon activation in the brain (Rice et al., 2012).

The role of ADAR1 in regulating type I interferon responses in the mammalian brain has not been comprehensively investigated, and the potential sources of dsRNA regulated by ADAR1 in glial cells are unknown. However, one possible source of dsRNA relevant to aging and AD is transposable elements (TEs). TEs, which include short and long interspersed nuclear elements (SINEs and LINEs), are non-coding repetitive DNA sequences that are often ignored as “junk DNA” (Bourque et al., 2018), but growing evidence links TE transcripts with inflammation, aging, and AD (Guo et al., 2018; Saleh et al., 2019; LaRocca et al., 2020; Ramirez et al., 2022; Wahl et al., 2023). While most work in this area has focused on the ability of select TE transcripts to form cytoplasmic DNA (another virus-associated molecular pattern) (Gorbunova et al., 2021), TEs also have the potential to form both inter- and intra-strand dsRNA (Porath et al., 2017). Recent data even show that TE-derived dsRNA induced by tau (a key pathological feature of AD) drives neuroinflammatory signaling in astrocytes (Ochoa et al., 2023).

Here, we extend on these recent findings by testing the hypothesis that ADAR1 inhibits pro-inflammatory signaling in human astrocytes, and that this may involve TE-derived dsRNA. Using a combination of transcriptomics (RNA-seq) and confirmatory molecular biology analyses, we show that ADAR1 suppression activates type I interferon and pro-inflammatory signaling pathways, and that this is associated with an increase in both dsRNA and TE transcripts with the potential to form dsRNA. We also provide evidence that our *in vitro* data may be clinically relevant, as we find reduced *ADAR1* gene expression with aging and AD, along with a similar increase in TE transcripts, in existing human brain transcriptome data. Collectively, our data suggest that ADAR1 may regulate TE-derived dsRNA that stimulates type I interferon/inflammatory signaling, and that reductions in ADAR1 could be a novel mechanism of neuroinflammation in aging/AD.

## Materials and methods

### Primary human astrocyte culture

Primary fetal human astrocytes (to rule out potential age/pathology-related variables) were used for all experiments. Cells were purchased from ScienCell and grown according to standard procedures at 37°C and 5% CO_2_ in a humidified incubator using astrocyte-specific medium and poly-L-lysine coated tissue culture plates. All cells had typical astrocyte morphology and tested >90% positive for GFAP expression at passage 1 (Supplementary Figure S1). Cells were subcultured at ~90% confluency and used at passages 3–6 for all experiments. No passage-associated differences were observed in any experiments.

### siRNA transfection

Astrocytes were transfected when 80% confluent using pre-designed siRNAs according to manufacturers’ instructions. Lipofectamine RNAiMAX reagent (ThermoFisher, 13,778–075) and siRNAs were diluted in Opti-MEM Medium (ThermoFisher, 31,985–070), combined and incubated for 5 min, then applied to cells in fresh medium at a final concentration of 15 nM. siRNAs used included: ADAR1 siRNA (ThermoFisher Silencer Select, s1008, target sequence: GAGAUUCUCUCAGCCUAAAtt); scramble siRNA (Santa Cruz Control siRNA A, sc36869, non-targeting sequence). Cells were incubated for 48 h after transfection to allow for knockdown to proceed. Media was then replaced with serum-free media and cells were incubated for an additional 24 h, after which cells were either: (1) rinsed with DPBS and frozen for immunoblotting or RNA isolation; or (2) fixed in 4% paraformaldehyde for immunofluorescence staining or fluorescence *in situ* hybridization. All cells were 90%–100% confluent for final analyses, and experiments were repeated at least three times, except for RNA-seq (one experiment with three replicates).

### Immunoblotting

Cell samples were thawed on ice and lysed using RIPA lysis buffer containing phosphatase and protease inhibitors (ThermoFisher/Roche). 5–10 ug of protein was separated by electrophoresis on 4–12% Bis-Tris gels (Bio-Rad) then transferred to nitrocellulose membranes (Bio-Rad). Membranes were blocked using 5% milk in TBS-Tween or 5% BSA in TBS-Tween (for phospho-antibodies) for at least 1–2 h at room temperature or overnight at 4°C. Primary antibodies were diluted in 5% milk in TBS-Tween or 5% BSA in TBS-Tween, added to membranes, and incubated overnight at 4°C. Antibodies used included: ADAR1 (Novus Biologicals, NBP3-05500, 1:1000), ICAM-1 (Novus Biologicals, NBP1-88700, 1:1000), MDA5 (Novus Biologicals, NBP1-76760, 1:500), phosphorylated (p)-IRF3 (ABclonal, AP0995, 1:1000), pNF-κB (ABclonal, AP0123, 1:1000), RIG-I (Novus Biologicals, NBP1-76732, 1:1000), and TNF-ɑ (Cell Signaling, 6,945 s, 1:500). Mouse and rabbit HRP-conjugated secondary antibodies (Cell Signaling, 7,076 s/7074 s, 1:2000) were diluted in 5% milk in TBS-Tween or 5% BSA in TBS-Tween, added to membranes, and incubated for 1 h at room temperature. Proteins were detected using ECL chemiluminescent substrate (Thermo) on a FluorChem E imager (ProteinSimple). Protein levels (ECL signal intensities) were measured using ImageJ2/Fiji and normalized to GAPDH (Novus Biologicals, 1:2000, NB100-56875).

### RNA isolation, sequencing, and bioinformatics

Cells were lysed with Trizol (Zymo Research), and RNA was isolated using an RNA-specific spin column kit (Direct-zol RNA MicroPrep, Zymo Research, R2062) including a DNase I treatment according to the manufacturer’s instructions. Ribosomal RNA-depleted total RNA libraries were generated using Zymo Total RNA library preparation kits, then sequenced on an Illumina NovaSeq6000 instrument to generate >60 M 151-bp paired-end fastq reads per sample. Reads were trimmed using fastp (v0.20.0) (Chen et al., 2018) and aligned to the hg38 genome using STAR (v2.7.3a) with default settings (Dobin et al., 2013). Differential gene expression from the gene counts generated by STAR was analyzed using DESeq2 (v1.30.1) (Love et al., 2014), and differentially expressed genes with an adjusted *p*-value (FDR) < 0.1, the standard DESeq2 cutoff, were used for gene ontology analyses in the g:Profiler program (Raudvere et al., 2019). TEs were identified using RepeatMasker annotation files and the RepEnrich2 program with default settings (Criscione et al., 2014), and differential expression of TEs was analyzed using DESeq2 with sample-specific size factors to normalize for library size. RNA editing was measured using SPRINT (Zhang et al., 2017) with default parameters. To determine RNA edits within TEs, SPRINT output files were intersected with the human RepeatMasker annotation file. Changes in editing in response to ADAR1 knockdown (i.e., “∆-edits”) were calculated by subtracting the number of edits per TE in ADAR1 siRNA-treated cells from the number of edits per TE in scramble siRNA-treated cells. RNA secondary structures were generated using the RNAfold Webserver (v2.5.1) (Lorenz et al., 2011). All data can be found on the Gene Expression Omnibus (GEO) website under accession number: GSE225369.

### Immunofluorescence staining

Cells were cultured on poly-L-lysine-coated glass chamber slides (Nunc Lab-Tek). After fixation in 4% paraformaldehyde for 10 min, cells were washed with DPBS, permeabilized with 0.25% Triton-X and for 10 min, washed again and then blocked using 3% normal goat serum/3% fetal bovine serum (Jackson ImmunoResearch) in DPBS for 30 min. Primary antibodies were prepared in 0.1% tween/5% normal goat serum in DPBS and added to cells to incubate overnight at 4°C. Primary antibodies used included: J2 (Novus Biologicals, NBP3-11395, 1:500) and MDA5 (Novus Biologicals, NBP1-76760, 1:500). After overnight incubation, cells were washed with DPBS and mouse/rabbit fluorescent probe-conjugated secondary antibodies (Invitrogen, A32731/A32727) diluted in 0.1% tween/5% normal goat serum were added to incubate for 1 h at room temperature in the dark. Cells were washed with DPBS, mounted using Prolong mounting medium containing DAPI (ThermoFisher, P36935), and allowed to set overnight at room temperature. Images were generated using an EVOS M7000 fluorescence microscope at 40x magnification, and per cell fluorescence was measured in five images per slide well for each treatment and normalized to the mean of all conditions using ImageJ/Fiji software, as previously reported (LaRocca et al., 2021).

### ELISA

Cell culture media was collected following the switch to serum-free media. To measure levels of CXCL10, media samples were diluted 1:20 and analyzed using a sandwich ELISA according manufacturer’s instructions (ABclonal, Ab83700). IFNβ was measured using undiluted cell culture media samples and a sandwich ELISA according manufacturer’s instructions (R&D Systems, DIFNB0).

### Fluorescence *in situ* hybridization

Fluorescence *in-situ* hybridization (FISH) probes for the transposable element L1MA6, a LINE with sequence similarity to several other L1 elements (to increase signal detection), were designed using Biosearch Technologies Stellaris Probe Designer Version 4.2, and 12.5 μM FISH probe stock solutions were prepared in TE buffer (10 mM Tris–HCL, 1 mM EDTA, pH 8.0). Cells were washed with DPBS and fixed with 4% formaldehyde in RNase-free DPBS for 10 min at room temperature, then washed again and permeabilized in 70% ethanol for 2 h at 4°C. After permeabilization, cells were washed, and RNA FISH probe in hybridization buffer along with primary antibodies (same dilutions as above for immunofluorescence) was added and incubated for 16 h at 37°C in the dark. Cells were washed again for 30 min at 37°C in the dark, then incubated for 30 min with 5 ng/mL DAPI in wash buffer before a final 5 min wash per manufacturer’s instructions. Slides were mounted using Prolong mounting medium (ThermoFisher) and imaged on an EVOS M7000 fluorescence microscope at 40x magnification.

### RNA-seq secondary analysis

Existing RNA-seq data used in this study are available via the GEO accession number GSE153875. Bioinformatics analyses of these data were performed as described above. Raw counts were normalized using size factors generated from DESeq2 for downstream statistical analyses.

### Statistical analyses

GraphPad Prism software was used for ANOVAs with post-hoc *t*-tests and unpaired *t*-tests for all experimental data, as well as normality testing and Pearson correlation analyses of RNA-seq data and chi-square analyses of increased versus decreased TE transcripts. DESeq2 was used for differential expression analyses as described above.

## Results

**Figure 1:**
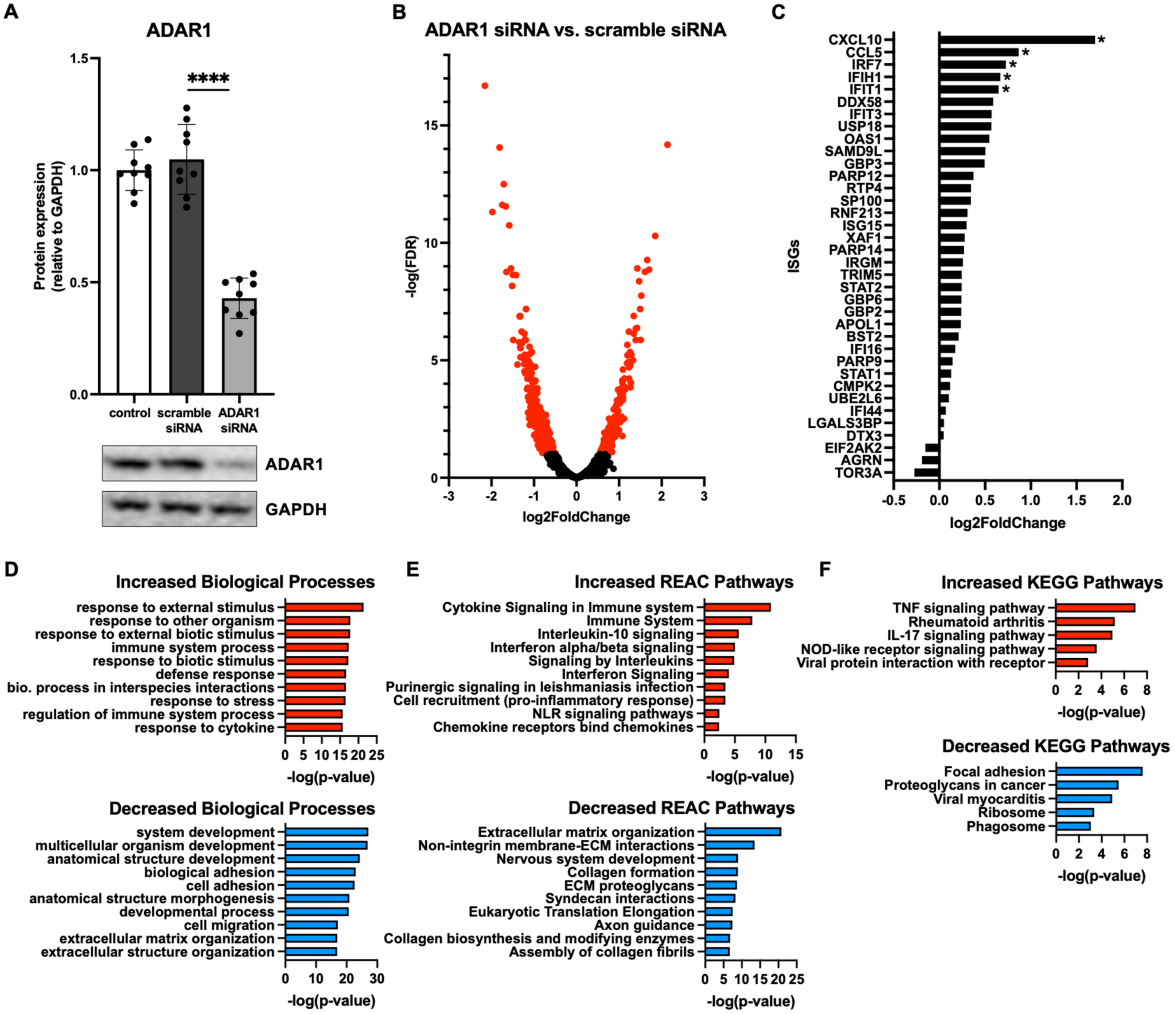
ADAR1 knockdown increases interferon and pro-inflammatory gene expression. **(A)** Immunoblot data showing knockdown of ADAR1. ****p ≤ 0.0001, one-way ANOVA, *F* = 104.3, DF = 26; *n* = 9/condition. Error bars represent standard deviation (SD). **(B)** Volcano plot of RNA-seq data for protein coding transcripts in astrocytes transfected with ADAR1 siRNA compared to scramble siRNA. Genes significantly increased/decreased in red (FDR < 0.1); *n* = 3/condition. **(C)** Log2FoldChange of interferon stimulating genes (ISGs) and genes related to interferon signaling. *FDR < 0.1. Gene list derived from Dhir et al. (2018). **(D-F)** Top 10 increased/decreased **(D)** GO biological processes **(E)** REAC and **(F)** KEGG pathways with ADAR1 knockdown compared to scramble siRNA.

ADAR1 is a key regulator of cytoplasmic dsRNA and inflammatory signaling activation in many tissues, but its role and potential dsRNA targets in human astrocytes have not been comprehensively investigated. Therefore, we knocked down ADAR1 by siRNA transfection in primary human astrocytes and performed transcriptomics (RNA-seq) to broadly profile resulting changes in gene expression. We were able to achieve a knockdown efficiency of ~60% (Figure 1A; Supplementary Figure S2A), and differential gene expression analyses showed that ADAR1 knockdown resulted in 220 increased and 745 decreased RNA transcripts compared to a scramble siRNA control (Figure 1B). Many increased transcripts were interferon stimulating genes (ISGs) or related to interferon signaling pathways (Figure 1C). Although only several of these genes/transcripts were increased at FDR < 0.1, compared to average gene expression, ISGs as a group were significantly increased (Supplementary Figure S2B), consistent with other studies of dsRNA-induced interferon activation (Dhir et al., 2018). Moreover, gene ontology analysis indicated that up-regulated biological processes with ADAR1 knockdown were related to immune responses and inflammation, whereas down-regulated biological processes were related to development and extracellular matrix organization (Figure 1D). We also found increased KEGG and REAC pathways reflecting pro-inflammatory and interferon signaling with ADAR1 knockdown, and decreased REAC pathways reflecting extracellular matrix organization (Figures 1E,F). These data show increases in pro-inflammatory/interferon-associated gene expression following ADAR1 knockdown, which is consistent with previous studies on ADAR1 suppression or dysfunction in other cell types (Guo et al., 2022a), and with astrocyte-related neuroinflammation (Hasel et al., 2021).

**Figure 2:**
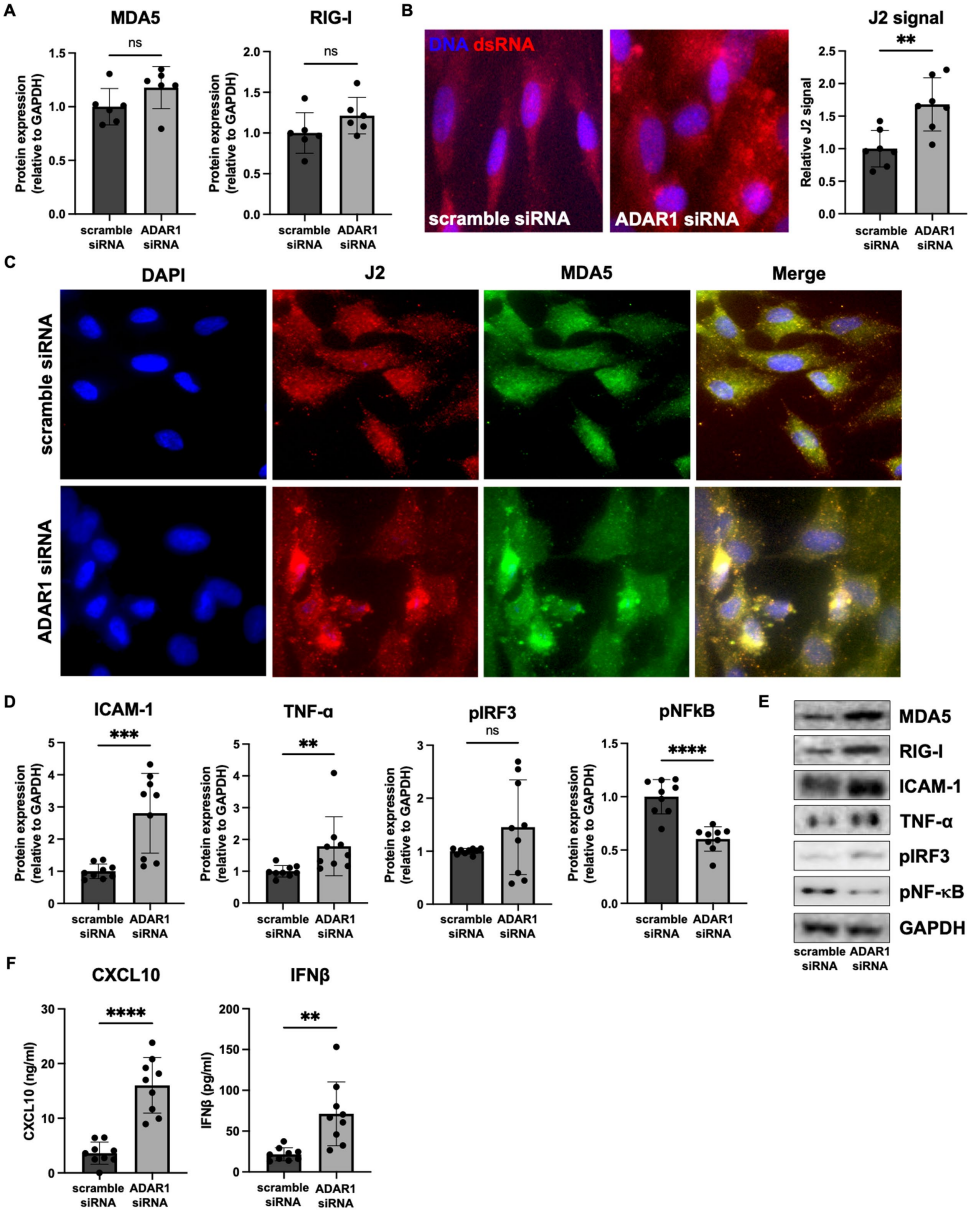
ADAR1 knockdown results in dsRNA increases, type I interferon signaling and pro-inflammatory protein expression. **(A)** Immunoblots for MDA5 and RIG-I following ADAR1 knockdown. Unpaired *t*-test; *n* = 6/condition. **(B)** Representative images and relative J2 (dsRNA antibody) immunofluorescence signal showing dsRNA accumulation following ADAR1 knockdown. **(C)** Representative immunofluorescence images showing co-localization of dsRNA (J2) and MDA5 after transfection with ADAR1 siRNA. **(D)** Summary immunoblot data for ICAM-1, TNF-α, pIRF3, pNF-κB, and **(E)** representative images following ADAR1 knockdown. **(F)** Concentrations of CXCL10 and IFNβ following ADAR1 siRNA knockdown. **p ≤ 0.01, ***p ≤ 0.001, ****p ≤ 0.0001 in unpaired *t*-test; *n* = 9/condition. Error bars represent SD.

In line with other reports (Liddicoat et al., 2015; Guo et al., 2021, 2022a), our RNA-seq data also showed that the dsRNA sensors MDA5 (*IFIH1* gene) and retinoic acid-inducible gene I (RIG-I; *DDX58* gene) were increased and borderline increased (FDR = 0.11), respectively, following ADAR1 knockdown (Figure 1C). Increases in these transcripts translated to modestly but not significantly elevated protein levels (Figures 2A,E) that tended to correlate with downstream products of MDA5/RIG-I signaling (*p* = 0.02–0.09, Supplementary Figure S3B). Moreover, consistent with the key role of these proteins in dsRNA sensing and the idea that their protein levels are not as important as their activity, we found that: (1) ADAR1 knockdown resulted in an increase in dsRNA (Figure 2B) and (2) although sensors like MDA5 were diffusely expressed in cells, they tended to colocalize with ADAR1 knockdown-induced dsRNA (Figure 2C). Furthermore, these events were coupled with increases in protein markers of pro-inflammatory astrocyte activation. For example, ADAR1 knockdown resulted in increases in intracellular adhesion molecule I (ICAM-1) and tumor necrosis factor α (TNF-α) (Figures 2D,E). We also found increases in secreted C-X-C motif chemokine ligand 10 (CXCL10) and interferon β (IFNβ), which are downstream products of MDA5/RIG-I stimulated type I interferon signaling, in astrocyte media after ADAR1 knockdown (Figure 2F). Furthermore, although not significant, we found a variable increase (including some highly activated cells/samples) in phosphorylated interferon regulatory factor 3 (pIRF3), a transcription factor downstream of MDA5/RIG-I involved in the production of CXCL10 and IFNβ (Figures 2D,E). These pIRF3 levels correlated positively with CXCL10 secretion (Supplementary Figure S3B), and a transcription factor motif analysis showed that multiple IRFs were associated with the gene expression differences we observed in response to ADAR1 knockdown (Supplementary material). Additionally, ADAR1 knockdown resulted in a decrease in phosphorylated nuclear factor kappa B (pNF-κB) (Figures 2D,E), which is similar to what others have reported (Garcia-Gonzalez et al., 2022), further supporting the role of IRFs in response to ADAR1 suppression. Thus, our data support the idea that, similar to other tissues, the primary response to ADAR1 knockdown in human astrocytes is type I interferon signaling driven by MDA5 and/or RIG-I binding to dsRNA.

**Figure 3:**
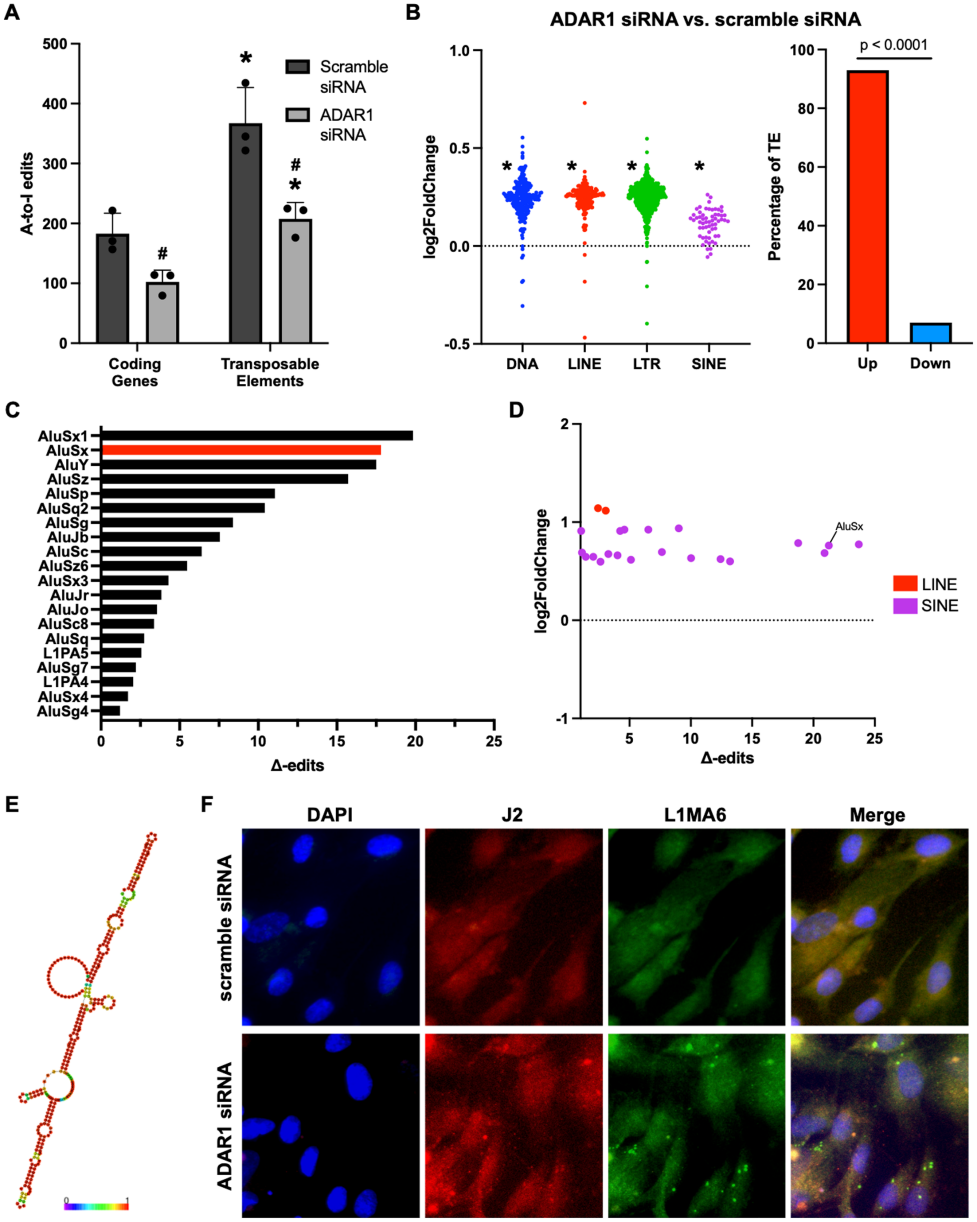
ADAR1 knockdown increases dsRNA-prone TE transcript accumulation. **(A)** Adenosine-to-inosine editing in coding genes and TEs with ADAR1 knockdown. *p ≤ 0.05 coding genes vs. TE and #*p* ≤ 0.05 ADAR1 siRNA vs. scramble siRNA; unpaired *t*-test; *n* = 3/condition. Error bars represent SD. **(B)** Changes in TE transcript expression by major TE type (log2FoldChange) and total TEs (% increased vs. decreased). *p ≤ 0.0001; Chi-squared test vs. mean gene expression log2FoldChange. **(C)** Top 20 Δ-edited TEs (average scramble siRNA edits – average ADAR1 siRNA knockdown edits). **(D)** Scatter plot showing highly edited (Δ-edits > 1) and highly expressed TE transcripts. **(E)** Potential RNA secondary structure of an increased, highly edited Alu element (AluSx). Red base-pairs indicate high base-pairing probabilities. **(F)** Fluorescence *in situ* hybridization images showing co-localization of dsRNA (J2) and LINE RNA after transfection with scramble siRNA or ADAR1 siRNA.

To determine the potential sources of dsRNA that accumulates after ADAR1 knockdown, we examined our RNA-seq data for A-to-I RNA editing (a key function of ADAR1 and an indirect, computational estimate of dsRNA). After ADAR1 knockdown, we found a ~50% reduction in A-to-I editing (Figure 3A), and most of these editing differences occurred in TEs rather than protein-coding genes, consistent with observations of editing in general (Porath et al., 2017). Importantly, reduced TE editing with ADAR1 knockdown was associated with increased expression of transcripts from four main types of TEs, and also with total levels of TE transcripts (Figure 3B; Supplementary Figure S4A), suggesting that ADAR1 is important for suppressing TE transcript accumulation. To determine which TE transcripts may accumulate and form dsRNA that is edited by ADAR1, we identified TE transcripts whose editing levels decreased (“∆-edits”) most with knockdown (i.e., presumably because they are usually heavily edited by ADAR1). We also examined changes in expression of these ADAR1-edited TE transcripts, and we found that many of the TE transcripts that both increased following knockdown and had high ∆-edits were SINEs, specifically Alu elements (Figures 3C,D), which are established ADAR1 targets. Moreover, several particularly likely Alu element ADAR1 targets, such as AluSx, had the potential to form highly intra-strand dsRNA structures (Figure 3E), and several of the most increased TE transcripts with ADAR1 knockdown had similarly dsRNA-prone structures (Supplementary Figure S4B). Additionally, a few LINEs, which are among the most abundant TEs in the human genome and have also been reported as ADAR1 targets, were increased and highly edited. Based on this observation and because Alu elements are relatively short and difficult to probe for using microscopy, we used FISH to confirm that LINE transcripts (longer and more amenable to FISH) colocalized with dsRNA immunofluorescence (Figure 3F). Together, these data support the idea that TEs, particularly SINEs and LINEs, may be an important endogenous source of dsRNA in astrocytes, and that ADAR1 is important for reducing TE-derived dsRNA.

**Figure 4:**
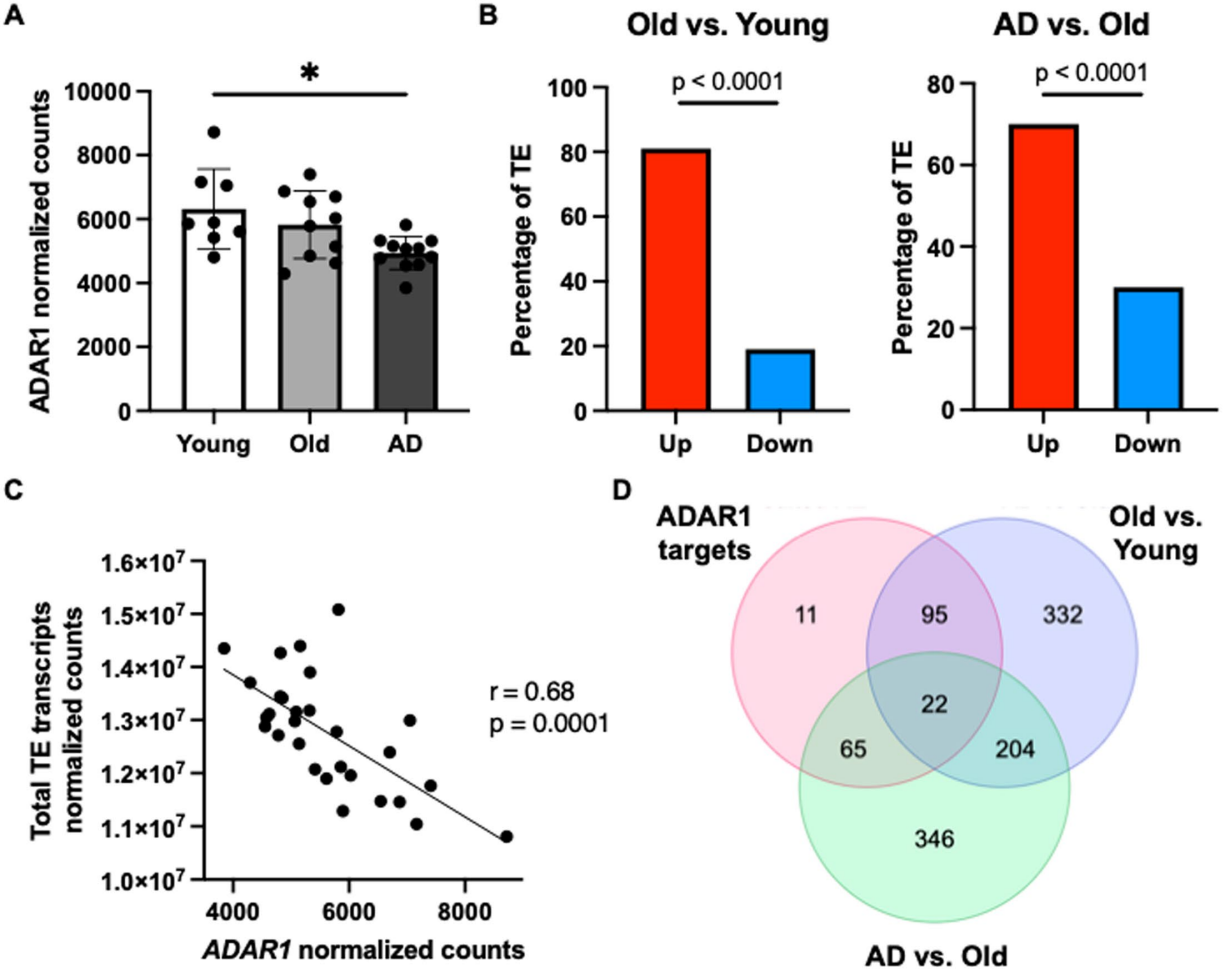
*ADAR1* gene expression declines with age/AD are associated with TE transcript accumulation in humans. **(A)** Changes in *ADAR1* gene expression with age and AD in postmortem brain samples from young (*n* = 8, average age 52 years), old (*n* = 10, average age 68 years), and Alzheimer’s disease (*n* = 12, average age 68 years) subjects, analyzed in Nativio et al. (2020). *p ≤ 0.05, one-way ANOVA, *F* = 5.113, DF = 28. **(B)** Differences in TE transcript expression by major TE type in older vs. younger and AD vs. older subjects. *p ≤ 0.0001; Chi-squared test vs. mean gene expression log2FoldChange. **(C)** Negative correlation between total TE transcript levels and *ADAR1* counts in all subjects (Pearson *r*- and *p*-values). **(D)** Similar TE transcripts among potential ADAR1 targets (high Δ-edits) and increased TE transcripts (> average log2FoldChange) in older vs. younger and AD vs. older subjects.

Finally, to determine if our data showing that ADAR1 knockdown in astrocytes results in innate immune sensor activation, type I interferon/pro-inflammatory signaling, and TE transcript accumulation might be clinically relevant, we conducted a secondary analysis of a published RNA-seq dataset on human brain tissue (Nativio et al., 2020) (chosen specifically for data quality and carefully matched subject groups). We found a significant reduction in overall *ADAR1* gene expression in AD patients compared to healthy young adults (Figure 4A), and although there was only a trend for lower *ADAR1* gene expression in older compared to younger adults, we did find an increase in TE transcript expression in the brains of these same subjects that was exacerbated in AD vs. healthy older adult brains (Figure 4B). In addition, we found significant/borderline significant correlations among age and some dsRNA-prone TEs that were also particularly increased with ADAR1 knockdown in our *in vitro* data (Supplementary Figures S5A,B). We also found a negative correlation between *ADAR1* counts and overall TE transcript levels in all of these subjects (Figure 4C), consistent with the idea that ADAR1 may be an important regulator of TE-derived dsRNA. Finally, in support of the idea that these events may contribute to neuroinflammation *in vivo*, among the most increased TE transcripts in AD vs. healthy older adults and older vs. younger adults, we found 22 TE transcripts that were also identified as ADAR1 targets (TE transcripts with high number of Δ-edits) in our *in vitro* analyses (Figure 4D). These TE transcripts largely consisted of LINEs and long terminal repeats (LTRs), whereas ADAR1-edited SINEs were more common in AD vs. healthy older adult brains (Supplementary material). Together, these data provide evidence of a role for ADAR1 in human brain aging and AD, and for the idea that TE-derived dsRNA may be an important, neuroinflammation-related ADAR1 substrate *in vivo*.

## Discussion

Pro-inflammatory signaling in glial cells is centrally involved in neuroinflammation, brain aging, and AD (Colombo and Farina, 2016; Matias et al., 2019; Giovannoni and Quintana, 2020). Here, we provide evidence that ADAR1 suppression causes interferon-associated pro-inflammatory signaling in human astrocytes, perhaps via TE-derived dsRNA, and that *ADAR1* gene expression is reduced while dsRNA-prone TE transcripts are increased with age and AD in the human brain. Collectively, our results suggest a potentially novel, endogenous mechanism for neuroinflammation with aging and disease, which could provide insight into future therapeutic targets.

Neuroinflammation, driven by glial cells like astrocytes, is a central mechanism of both brain aging and AD, and type I interferon signaling has been established in age- and AD-related neuroinflammation (Taylor et al., 2018; Roy et al., 2020). Others have shown that reductions in ADAR1 may be a driver of interferon signaling in various tissues, but the transcriptomic effects of ADAR1 suppression in human astrocytes have not been extensively studied. Our data show that the accumulation of endogenous dsRNA due to ADAR1 knockdown results in increased expression of genes/pathways related to type I interferon and pro-inflammatory signaling in human astrocytes, which is consistent with previous reports in the brain and in other cell types (Yang et al., 2014; Liddicoat et al., 2015; Deng et al., 2020; Guo et al., 2021, 2022b). Our data also show that ADAR1 knockdown leads to downregulation of biological processes and pathways related to extracellular matrix organization in astrocytes, events that have been linked with changes synaptic plasticity, response to injury, and impaired blood brain barrier integrity (Faissner et al., 2010; Baeten and Akassoglou, 2011; Johnson et al., 2015). We also found a downregulation of processes related to development with ADAR1 knockdown, which may reflect the connection between ADAR1 and cellular development (Wang et al., 2004; Guo et al., 2021, 2022b).

Type I interferon and pro-inflammatory signaling can occur as a result of multiple upstream stimuli (e.g., injury, infection). Here, we show that type I interferon and pro-inflammatory signaling resulting from ADAR1 knockdown is likely driven by cytoplasmic dsRNA binding to MDA5 and/or RIG-I, as opposed to more generic inflammation-related pathways like NF-κB signaling. This observation is in line with previous work showing that NF-κB is downregulated to prevent apoptosis following ADAR1 suppression, and that pro-inflammatory signaling in this context occurs through interferon regulatory factor 7 (IRF7; an additional downstream transcription factor of MDA5/RIG-I) (Garcia-Gonzalez et al., 2022). Our findings are also consistent with studies in other tissues in which gene silencing/transgenic approaches have been used to show that ADAR1 regulates cytoplasmic dsRNA levels, and that the inflammatory response to this dsRNA occurs through the MDA5/RIG-I signaling pathway (Yang et al., 2014; Liddicoat et al., 2015; Guo et al., 2022a,b). We observed only transcript (not protein) increases in these proteins, but we found that MDA5 colocalized with dsRNA (the key event required for signaling via this pathway). Additionally, we note that our RNA-seq data show that other dsRNA sensor proteins (e.g., PKR, OAS1) were not significantly increased with ADAR1 knockdown, further supporting a specific role for MDA5/RIG-I in interferon and pro-inflammatory signaling with ADAR1 suppression. Our results are limited by our *in vitro* approach, as neuroinflammation is a physiological response that cannot be fully measured in one cell type. *In vivo*, *Adar* mutant *Drosophila* and *Adar1* mouse AGS models have been used to show that dysfunctional ADAR1 results in innate immune activation in brain-like tissue and mammalian brains, respectively (Deng et al., 2020; Guo et al., 2021; Inoue et al., 2021). Future work using additional *in vivo* models will be important to determine if similar events may occur in brain aging and AD, but null *Adar1* knockouts are embryonically lethal (Wang et al., 2004). As such, recent work developing mouse strains with mutations in the catalytic domains of *Adar1* that decrease dsRNA editing and stimulate interferon signaling may be particularly promising (Guo et al., 2021, 2022b). However, recent data also suggest that ADAR1 downregulation can influence age-related processes like senescence independent of RNA editing (Hao et al., 2022). Therefore, it may be necessary to develop an *in vivo* model of ADAR1 overexpression to fully investigate its protective effects.

Importantly, there are many potential triggers for neuroinflammation that involve external causes (e.g., viral infection), non-CNS processes (e.g., peripheral inflammation), and/or CNS signals in response brain aging or AD pathology (e.g., amyloid beta accumulation) (Qin et al., 2007; Li et al., 2018; Klein et al., 2019; Roy et al., 2020). However, our data suggest a novel endogenous cause of neuroinflammation involving ADAR1 reductions and subsequent TE-derived dsRNA accumulation. These findings may be particularly important, as we and others have shown that TE transcripts increase with aging and AD (Guo et al., 2018; LaRocca et al., 2020; Wahl et al., 2023) which, in combination with reduced ADAR1 activity, would support the accumulation of TE-derived dsRNA (Kassiotis and Stoye, 2016; Chen and Hur, 2022). Indeed, we found a global increase in total TE transcripts after ADAR1 knockdown *in vitro*, and the associated reduction in A-to-I editing occurred mostly in repetitive sequences as compared to protein coding genes, which is consistent with other reports (Porath et al., 2017). We also identified highly expressed TE transcripts that are targets of ADAR1 (and therefore likely to form dsRNA), and these included SINEs (Alu elements) and LINEs (L1 elements). Alu and L1 elements are among the most common TEs in the human genome, and both have been shown to be edited by ADAR1 (Orecchini et al., 2017; Ahmad et al., 2018), in line with the idea that they may be an important source of dsRNA. To confirm these findings more rigorously, future studies should leverage RNA immunoprecipitation sequencing to identify the specific TE transcripts that increase with aging/AD and bind to ADAR1 in different CNS cell types, which could provide insight into the potential to therapeutically target these events.

Rigorous characterizations of ADAR1 TE targets in the future may be particularly important, because our analyses of published RNA-seq data (Nativio et al., 2020) suggest that our *in vitro* findings could be clinically relevant. We found reductions in *ADAR1* gene expression in older adult and AD patient brains, and a corresponding global increase in TE transcripts in both older adults and AD patients. In fact, higher *ADAR1* counts correlated with lower total TE transcript counts across aging and AD, supporting the idea that TEs may be an endogenous source of dsRNA that is regulated by ADAR1 in humans. Importantly, these analyses were based on bulk brain tissue containing multiple cell types, while our *in vitro* data were from homogenous astrocyte cultures. As such, it remains to be determined if astrocytes are the main cell type involved in ADAR1-related inflammatory signaling *in vivo*. However, we note that *ADAR1* counts are inversely correlated with *GFAP* counts in this dataset, suggesting a possible role for changes in ADAR1 expression in astrocytes in the whole brain. Additionally, pro-inflammatory CNS cell activation with aging and AD is also associated with other cellular/transcriptomic processes (e.g., increased chromatin accessibility of IRFs, DNA damage, and senescence) (Yu et al., 2015; Frisch and MacFawn, 2020; Rasa et al., 2022), and it will be important to carefully design future studies on this topic to take these into account (e.g., by using multi-omics techniques to rule out other potential cell types or drivers of neuroinflammation). Finally, one key question is: What drives reductions in ADAR1 with age and/or AD? ADAR1 is reported to increase in various types of cancer (Xu and Öhman, 2018), and cancer risk increases with age (Berben et al., 2021). As such, it is possible that cells downregulate ADAR1 with age to prevent excessive editing that may contribute to cancer development, which would be consistent with the idea of antagonistic pleiotropy as a key mechanism of aging (Austad and Hoffman, 2018). Therefore, before ADAR1-targeted therapeutic strategies can be advanced, long-term (e.g., lifespan) studies may be required to investigate the direct effects of ADAR1 *in vivo* on age- and AD-related neuroinflammation, as well as other health outcomes.

Collectively, our data suggest a critical role for ADAR1 in neuroinflammation via type I interferon activation in human astrocytes. Type I interferon is increasingly linked with neuroinflammation in brain aging and AD, and our findings point to a novel, upstream mechanism that may activate it (i.e., TE-derived dsRNA as a result of reduced ADAR1). The specific TE transcripts that form cytoplasmic dsRNA and activate type I interferon signaling remain unknown, but our pilot studies may serve as a foundation for future work investigating ADAR1 in brain aging and AD.

## Data availability statement

The datasets presented in this study can be found in online repositories. The names of the repository/repositories and accession number(s) can be found in the article/Supplementary material.

## Ethics statement

Ethical approval was not required for the studies on humans in accordance with the local legislation and institutional requirements because only commercially available established cell lines were used (obtained from ScienCell) and the transcriptome information used in secondary analyses was extracted from a publicly available database (https://www.ncbi.nlm.nih.gov/geo/).

## Author contributions

CM: Conceptualization, Data curation, Formal analysis, Writing – original draft, Writing – review & editing. AC: Data curation, Writing – review & editing. TL: Conceptualization, Data curation, Formal analysis, Funding acquisition, Writing – review & editing.

## Figures and Tables

**Table 1:** 

AD	Alzheimer’s disease
ADAR1	adenosine deaminase acting on RNA 1
AGS	Aicardi Goutières syndrome
A-to-I	adenosine-to-inosine
CNS	central nervous system
CXCL10	C-X-C motif chemokine ligand 10
dsRNA	double-stranded RNA
FISH	fluorescence *in-situ* hybridization
GEO	gene expression omnibus
ICAM-1	intracellular adhesion molecule I
IFNβ	interferon β
IRF7	interferon regulatory factor 7
ISGs	interferon stimulated genes
KEGG	Kyoto Encyclopedia of Genes and Genomes
LINEs	long interspersed nuclear elements
LTRs	long terminal repeats
MDA5	melanoma differentiation-associated protein 5
pIRF3	phosphorylated interferon regulatory factor 3
pNF-κB	phosphorylated nuclear factor kappa B
REAC	Reactome
RIG-I	retinoic acid-inducible gene I
SD	standard deviation
SINEs	short interspersed nuclear elements
TE	transposable elements.
TNF-α	tumor necrosis factor α
