# Supplementary material for: ADAR1 suppression causes interferon signaling and transposable element transcript accumulation in human astrocytes
Source: Front Mol Neurosci. 2023 Oct 25;16:1263369. doi: 10.3389/fnmol.2023.1263369 (PMC10685929; doi:10.3389/fnmol.2023.1263369)
Supplement: Supplementary file 1 [file Data_Sheet_1.docx]

**Supplementary Material**

**Excel File S1** RNA-seq data in support of all findings. Spreadsheet tabs include: **A** Gene DESeq2 output for ADAR1 siRNA vs. scramble siRNA. **B** Significantly upregulated genes (FDR < 0.1; log2FoldChange > 0) and gene ontology analysis. **C** Significantly downregulated genes (FDR < 0.1; log2FoldChange < 0) and gene ontology analysis. **D** TE DESeq2 output for ADAR1 siRNA vs. scramble siRNA. **E** A-to-I edited TEs with corresponding Δ-edits. **F** TE DESeq2 output for older vs. young adults. **G** TE DESeq2 output for AD vs. older adults. **H** List of TEs per section of Venn diagram in Figure 4.


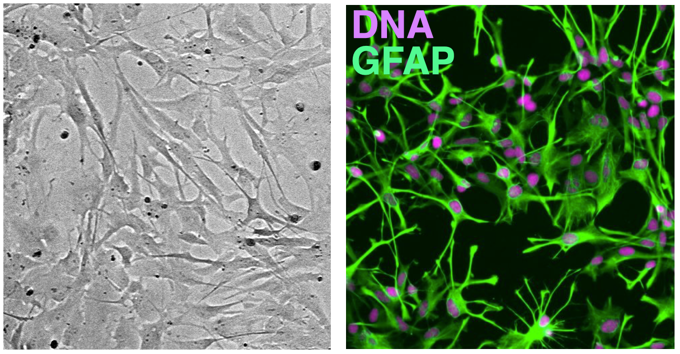


**Supplemental Figure 1.** Representative image of commercially obtained primary astrocytes used for all experiments. Left: Phase contrast image of typical astrocyte morphology in the current experiments. Right: positive staining for glial fibrillary acidic protein (GFAP, example courtesy ScienCell).

**Supplemental Figure 2.** ADAR1 knockdown increases interferon stimulated gene expression. **A** Immunoblots for ADAR1 in control, scramble siRNA, and ADAR1 siRNA transfected cells. **B** Increase in average interferon stimulated gene expression with ADAR1 knockdown (mean log2FoldChange vs. that of all other genes). ***p ≤ 0.001, Mann-Whitney test.

**Figure S3**

**Supplemental Figure 3.** Immunoblots showing increases/decreases in proteins following ADAR1 knockdown and correlations among MDA5, RIG-I, pIRF3 protein expression and CXCL10. **A** Immunoblots of dsRNA sensors and pro-inflammatory astrocyte markers after ADAR1 knockdown. **B** Correlations between MDA5, RIG-I, and pIRF3 protein expression and CXCL10 concentration. p-values and r values calculated using a simple linear regression.

**Figure S4**

**Supplemental Figure 4.** Increase in TE transcripts that form intra-stranded dsRNA following ADAR1 knockdown. **A** MA plot showing general increase in TE transcript expression with ADAR1 knockdown. Genes shown in grey, TE transcripts shown in black. Red dashed line indicates shift in average TE transcript log2FoldChange. p < 0.0001, Chi-squared test. **B** Potential RNA secondary structures of highly increased TE transcripts with ADAR1 knockdown. Red base pairs indicate high base-pairing probabilities.

**Figure S5**

**Supplemental Figure 5.** Correlations between age and dsRNA-prone TE transcripts that were highly increased with ADAR1 knockdown. **A** Positive correlation between UCON62 counts vs. age at death, and potential RNA secondary structure of UCON62. **B** Positive correlation between Eulor2C counts vs. age at death, and potential RNA secondary structure of Eulor2C. p-values and r-values calculated using simple linear regression. RNA-seq data derived from Nativio et al., 2020.
